# Reply to: Off-label use of repurposed ivermectin for SARS-Cov-2 infection should be banned by authorities unless efficacy is proven

**DOI:** 10.51866/lte.671

**Published:** 2024-07-17

**Authors:** Abdul Jalil Aina Amanina, Tar'ali Filza Nur Athirah

**Affiliations:** 1 BPharm, MClin Pharm, Faculty of Pharmacy and Health Sciences, Royal College of Medicine Perak, Universiti Kuala Lumpur, Ipoh, Perak, Malaysia. Email: aina.amanina@unikl.edu.my; 2 BPharm, Faculty of Pharmacy and Health Sciences, Royal College of Medicine Perak, Universiti Kuala Lumpur, Ipoh, Perak, Malaysia.

**Keywords:** Attitude, COVID-19, Ivermectin, Knowledge, Prevalence

**Dear editor,**

Thank you for the opportunity to address the concerns raised regarding our recent publication titled ‘Knowledge, attitude and prevalence of ivermectin use as coronavirus disease treatment: A cross-sectional study among a Malaysian population’.^1,2^

We appreciate the critical evaluation of our study design. Indeed, while electronic questionnaires offer convenience, we acknowledge their limitations, including the potential for inaccurate reporting and difficulty in confirming the identity of respondents. However, they also offer advantages such as ease of data collection and the potential for anonymity, which can encourage more candid responses. While electronic surveys have inherent limitations, they are cost-effective and facilitate easy data collection, particularly during the COVID-19 pandemic, when the data were collected and when in-person interactions were limited. Rigorous validation processes can help ensure the reliability of collected data. We agree that future research could benefit from a more robust methodology, such as employing a multicentre design to enhance the representativeness of the sample.

We acknowledge the limitation of our study’s single-centre design and modest sample size. While our findings provide valuable insights into the knowledge and attitudes of a subset of the Malaysian population, we agree that a multicentre study with a larger, more diverse sample would strengthen the generalisability of our conclusions. We hope that future research endeavours will address this concern by expanding the scope of the study population.

The questionnaire utilised in our study was indeed validated prior to its implementation. We apologise for the oversight in not providing detailed information on the validation process in our initial publication. We promptly address this by providing supplementary materials (Appendix A) outlining the validation methodology as a reference.

## Appendix A

Content validation was conducted to support the relevance, clarity and coherence of the questionnaire used. This involved expert judgements about the questionnaire content in relation to the defined domain of knowledge or performance of ivermectin use as COVID-19 treatment. A content validation form was prepared and distributed to two experts in their respective fields from Universiti Kuala Lumpur Royal College of Medicine Perak and Universiti Teknologi MARA Puncak Alam Campus. The experts were requested to critically review all domains and their items to check their relevance before scoring each item.

Each item was allocated with a relevance rating ranging from 1 to 4. Ratings of 4 and 3 were equivalent to 1, while ratings of 2 and 1 were equivalent to 0. The content validity index (CVI) must be at least 0.8 to meet the content validity requirement.^3^ The analysis revealed that the CVI for each item was >0.80, which indicated that it met the criteria Table 1)

**Table 1:** CVI among the three experts.

CVI	Expert 1+2+3
S-CVI/Ave	0.94
S-CVI/UA	0.83
Average proportion of items judged as relevant among the three experts	0.94

CVI, content validity index S-CVI/Ave, scale-level content validity index based on the average method S-CVI/UA, scale-level content validity index based on the universal agreement method

While our study primarily focused on assessing the level of knowledge, attitude and prevalence of ivermectin use among the participants, we did not specifically analyse the impact of vaccination status on ivermectin use. This decision was made to maintain the clarity and simplicity of our questionnaire design. However, we fully acknowledge the importance of vaccination status as a potential influencing factor of attitude and behaviour, particularly in the context of emerging COVID-19 treatment strategies. Future research endeavours should consider incorporating this aspect to provide a more comprehensive understanding of the dynamics surrounding ivermectin use in relation to vaccination status.

The potential side effects of ivermectin underscore the importance of cautious consideration when repurposing this drug for COVID-19 treatment. While our study did not directly investigate the reported side effects among the participants, we acknowledge the need for vigilance in monitoring adverse reactions associated with off-label ivermectin use. Any reports of side effects should be thoroughly documented and investigated to inform clinical decision-making and public health policies.

Regarding the predominance of students in our study population, we acknowledge the potential for selection bias and apologise for any ambiguity in our inclusion criteria. The inclusion of students may reflect accessibility to electronic surveys among this demographic rather than deliberate selection bias. Nonetheless, we recognise the importance of diversifying the study population to mitigate bias and ensure the robustness of our findings. Future studies should strive for greater demographic representation to enhance the validity and generalisability of results.

In conclusion, we appreciate the constructive feedback provided and acknowledge the limitations of our study. Addressing these concerns will undoubtedly strengthen the integrity and applicability of our findings. We remain committed to contributing to the scientific discourse surrounding COVID-19 treatment strategies and welcome further collaboration and inquiry in this endeavour.
